# Address to the Faculty of Pennsylvania and of the United States in General

**Published:** 1818

**Authors:** James Carver

**Affiliations:** Philadelphia


### ADDRESS.

TO

### THE FACULTY OF PENNSYLVANIA,

AND OF THE UNITED STATES GENERALLY.

GENTLEMEN,

THROUGH the great progress which has of late years been made in comparative anatomy, by the establishment of Veterinary Colleges throughout Europe, there is known to be so great a dissimilitude of parts in every species of the animal creation, that it cannot create surprise, to see me dedicate a Treatise on the Diseases of Horses and other Quadrupeds, to those who now stand so pre-eminent as judges of the diseases of mankind. I should not, however, have ventured to have made thus free with your names, if the great desire shown me by the Faculty of Philadelphia to advance my views in disseminating veterinary science, and the good intended in the following work, had not encouraged me to make this address.

Many of the advantages requisite to the obtainment of a skill in this branch of domestic science, have,

I flatter myself, been imbibed from my cradle. Born with a genius turned for studying the nature of horses, I possessed many advantages in early life from the experience of a father, who at that time was a breeder of running horses for most of the nobility in England, as also an experienced agriculturist. I likewise added considerably to my stock of knowledge before I began to travel, by an ardent application to the study of civil and military equitation. But not satisfied, and still irresolute what course to steer, I went to India, and placing myself under a celebrated veterinarian, who had been master of horse to the king, and who was then officiating as master of equitation and veterinary surgeon to marquis Wellesley and the honourable Governor's body guards, I there acquired a very extensive knowledge of the horses and cattle of that country, and some additional information in veterinary practice. But owing to ill health, and various disappointments, I left India, and came to this country with an intention of introducing the art of equitation. Finding the country not sufficiently mature for its admittance, and the door of industry open to every man, I for a time turned my pursuits another way. At length arriving in Philadelphia, and being introduced to Dr. Benjamin Rush of this city, through the acquaintance of Mr. Bradford, attorney at law, my predilection for my present studies did not escape the observation of my late and much lamented friend, by whose advice, and the assistance of him, the honourable judge Peters, Dr. Caldwell, Dr. James, professor of midwifery, Dr. Cox, professor of chemistry, general Cadwallader, and the Agricultural Society, I, at the age of forty, turned school-boy to obtain this branch of knowledge scientifically, and proceeded to the *London Veterinary College*, where by the great pains I took to obliterate all the prejudices of my former studies, I hope that my exertions to advance the grand cause of humanity among the brute creation, will entitle me to lay some claim to the confidence and patronage of the public in the publication of this useful work. I have waded through many difficulties in endeavouring to disseminate a knowledge of the veterinary art throughout the union, and my various trials have been arduous and unceasing.

Having pursued my professional studies in England with industry and integrity, according to the plan I originally proposed; and having during my residence there refused a tempting offer of five hundred pounds a-year to go to India, and a still more tempting one of some thousands to go to Russia, I, through the advice of William Allen and John Capper, of the Society of Friends in England, returned to this country to lay the foundation stone of the veterinary art in Pennsylvania. I hope that the plan I have now fixed on to diffuse the science, will meet with a better reward than it hitherto has done. All I mean by this *new attempt* in travelling through the country on the plan proposed, is to endeavour to be useful in promulgating knowledge, and to contribute *my mite* towards the benefit of mankind and the brute creation.

I am, Gentlemen,

Most respectfully,

Your obliged and ob't servant,

JAMES CARVER.

*Philadelphia, July,* 1818.

